# Author Correction: Diurnal and circadian rhythmicity of the human blood transcriptome overlaps with organ- and tissue-specific expression of a non-human primate

**DOI:** 10.1186/s12915-022-01320-4

**Published:** 2022-05-06

**Authors:** Carla S. Möller-Levet, Emma E. Laing, Simon N. Archer, Derk-Jan Dijk

**Affiliations:** 1 Bioinformatics Core Facility, Faculty of Health and Medical Sciences, University of Surrey, Guildford, UK; 2 School of Biosciences and Medicine, Faculty of Health and Medical Sciences, University of Surrey, Guildford, UK; 3 Surrey Sleep Research Centre, Faculty of Health and Medical Sciences, University of Surrey, Guildford, UK; 4 UK Dementia Research Institute, Care Research and Technology Centre at Imperial College, London and the University of Surrey, Guildford, UK

**Author Correction to: BMC Biol 20, 63 (2022)**

**https://doi.org/10.1186/s12915-022-01258-7**

The original article [1] mistakenly swapped the figure images for Figs 17 and 18. This has since been corrected.
